# Effects of CD20+ B-cell infiltration into allografts on kidney transplantation outcomes: a systematic review and meta-analysis

**DOI:** 10.18632/oncotarget.16229

**Published:** 2017-03-15

**Authors:** Yingying Lu, Bingjue Li, Qixia Shen, Rending Wang, Zhimin Chen, Hong Jiang, Jianghua Chen

**Affiliations:** ^1^ Kidney Disease Center, The First Affiliated Hospital, College of Medicine, Zhejiang University, 310003, P.R. China

**Keywords:** CD20+ B cell, kidney transplantation, acute rejection, graft loss, steroid resistance

## Abstract

The effects of CD20+ B-cell infiltration during acute rejection on graft outcomes are controversial. The objective of this systematic review and meta-analysis was to clarify this issue. We performed a systematic literature search for studies published up to January 14, 2016. A total of 5 studies, with 200 patients, were included. The presence of CD20+ B cells in renal biopsies during allograft rejection was associated with graft loss and steroid resistance. No association of CD20+ B-cell infiltration with C4d-positive staining of the peritubular capillaries in renal biopsies was found in the analysis of patients who experienced kidney graft rejection. In conclusion, CD 20+ B cell infiltration during allograft rejection was associated with an increased risk of graft loss and steroid resistance.

## INTRODUCTION

Acute rejection can lead to chronic allograft nephropathy (CAN) and graft loss and can exacerbate organ shortages by resulting in re-transplantation [1]. Current immunosuppressive treatments for post-transplant management mainly focus on T-cell pathways. This has decreased the incidence of acute rejection. However, there is still a phenotype of acute rejection that is more recalcitrant to conventional treatment. This indicates that other mechanisms may be involved in this process.

Sarwal et al. detected B lymphocyte gene expression and CD20+ B-cell infiltration in kidney allograft biopsies, suggesting the potentially pathogenic role of B lymphocytes in acute renal rejection [2]. However, the relationship between CD20+ B cells and the clinical outcome of acute rejection remains controversial. Several studies support the idea that the infiltration of CD20+ B cells during allograft rejection is associated with higher serum creatinine levels, steroid resistance or poor graft survival [2–8]. In many other studies, however, no impact of CD20+ cells on refractory rejection or graft outcome has been found [9–13]. Furthermore, some studies have linked CD20+ B cells with a favorable clinical prognosis [14].

Therefore, we performed a meta-analysis to verify whether the presence of CD20+ B cells in renal biopsies could be a predictive marker for worse allograft outcomes after transplant rejection.

## RESULTS

### Literature search results and study characteristics

**Figure 1:**
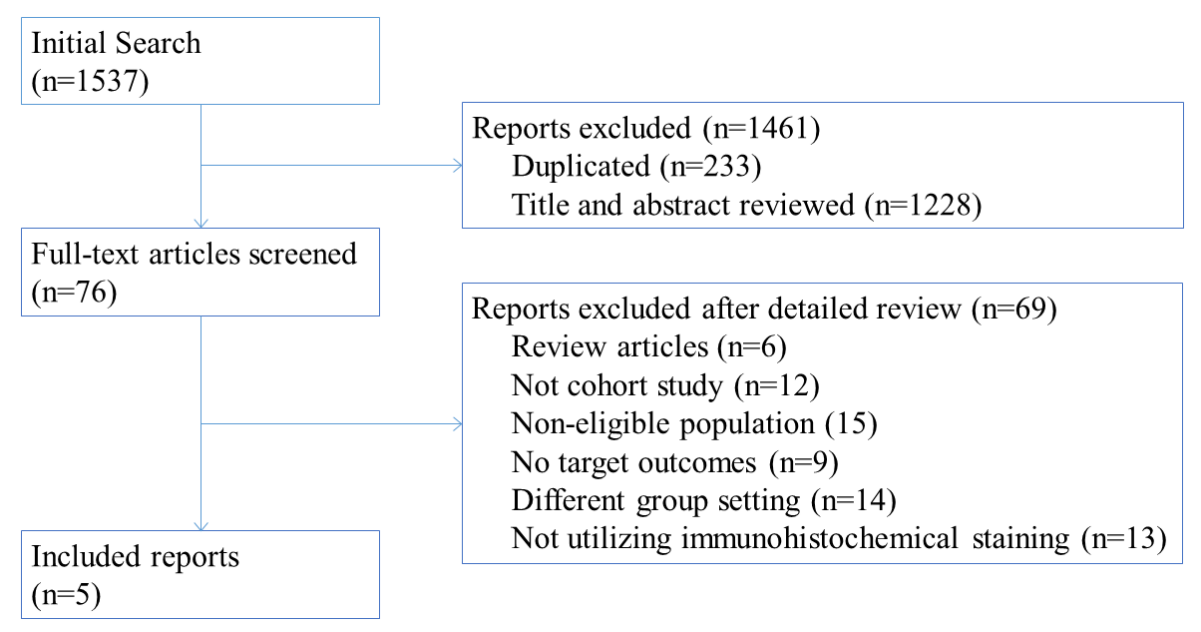
Flow chart of study selection

A total of 1537 potentially relevant citations were identified according to the search strategy. Of these citations, 1461 were excluded after their titles and abstracts were screened, leaving 76 studies for a full-text assessment. Examples of the potential citations that were rejected from the second screening are provided in Supplementary Table 3. Finally, a total of 5 eligible studies, including200 study subjects, were analyzed [2, 4, 6, 7, 9] (Figure 1).

The main characteristics of the included studies are presented in Table 1. Panel-reactive antibodies (PRAs) [4, 9], human leukocyte antigen (HLA) mismatches [6, 7], B-cell flow cytometric crossmatches (B-FCXM) [4, 7] and induction therapy [4, 7] were reported in two studies, whereas cold ischemia time [4] and donor specific antibodies (DSAs) [6] were reported in one study. Primary kidney disease was not described in any study. Baseline immunosuppression and rejection treatments were presented differently among the articles [4, 7, 9].

**Table 1:** Characteristics of the studies included in the meta-analysis

Study	Number of patients^a^	Age of biopsy (yr)^a^	Male(%)^a^	DCD(%)^a^	DGF(%)^a^	Time to AR (month)^a^	Follow-up (yr)^a^	Patients	Definition of CD 20 positive/negative^a^
Bagnasco et al . 2007	28/28	49 /44	75.0/60.7	NA	32.1/46.4	2.7/2.5	at least 4yr	first year ACR I-II(Banff 97)	≥100/HPF /<50/HPF
Hippen et al. 2005	6/21	40 /41	66.7/61.9	83.3/66.7	100.0/100.0	4.1/6.1	at least 4yr	first year ACR IA-IB(Banff 97)	strong and diffuse/trace or rare
Hwang et al. 2010	23/31	37 /40	69.6/48.4	4.3/22.6	8.7/12.9	NA	3.6/4.2	first time ACR I-II(Banff 97)	≥275/HPF /<100/HPF
Sarwal et al. 2003	9/22	NA	NA	NA	NA	NA	NA	ACR	≥275/HPF /<100/HPF
Zarkhin et al. 2008	17/15	14.5 /13	47.1/33.3	23.5/26.7	NA	51.9/26.1	5.8/5.2	AR	≥275/HPF /<100/HPF

DCD: deceased donor; DGF: delayed graft function; HPF: high power field; ACR: acute cellular rejection; AR: acute rejection; NA, not available

^a^: CD 20 positive group/CD 20 negative group

### Meta-analysis

#### C4d staining

**Figure 2:**
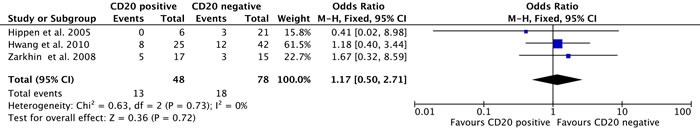
Meta-analysis of the incidence of C4d-positive staining between CD 20-positive and CD 20-negative groups Abbreviations: CI, confidence interval; KH, Knapp-Hartung method.

Three studies assessed the C4d staining of the peritubular capillaries in renal biopsies from 126 patients. There was no heterogeneity between the CD20-positive and CD20-negative groups (I^2^ = 0%; Pheterogeneity = 0.73). The fixed effects model was adopted, and no association was found between CD20+ B-cell infiltration and the C4d-positive staining of the peritubular capillaries during acute graft rejection (OR, 1.17; 95% CI, 0.50-2.71) (Figure 2).

### Steroid resistance

**Figure 3:**
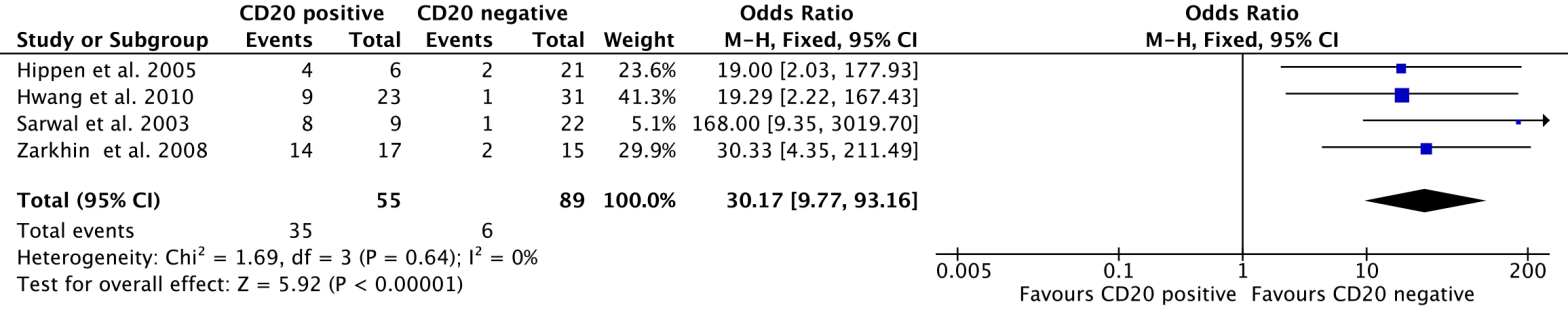
Comparison of the CD 20-positive group versus the CD 20-negative group for the incidence of steroid-recalcitrant graft rejection Abbreviations: CI, confidence interval; KH, Knapp-Hartung method.

Steroid resistance was evaluated in 4 studies including 144 patients. In Hwang et al., 2010 and Hippen et al., 2005, acute rejection was treated with 3-4 daily boluses of intravenous methylprednisolone (500 mg/day), followed by a 5-7 days’ oral steroid taper. If steroid resistance occured, additional treatment with antithymocyte globulin or muromonab-CD3 (OKT3) was given. However, the steroid doses weren't given in two other studies. An association between CD20+ B-cell infiltration and steroid resistance after transplant rejection (OR, 30.17; 95% CI, 9.77-93.16; I^2^ = 0%; Pheterogeneity = 0.64) was found (Figure 3). CD20+ B-cell infiltration might account for the need for more courses of steroids for the treatment of rejection.

### Graft loss

**Figure 4:**
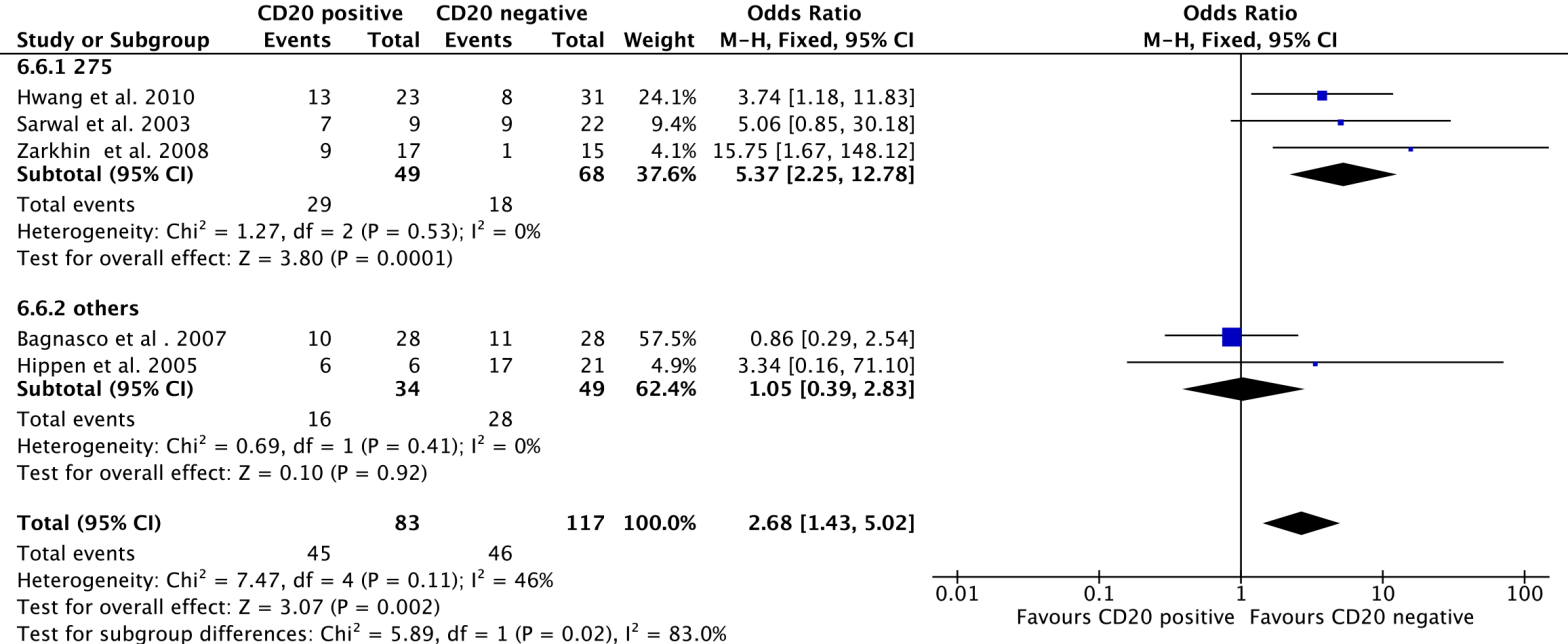
Meta-analysis of graft loss incidence between the CD 20-positive and CD 20-negative groups and subgroup analysis of studies based on different CD 20-positive definitions Abbreviations: CI, confidence interval; KH, Knapp-Hartung method.

When all 5 studies, including 200 patients, were assessed, CD20+ B cell infiltration during rejection was associated with an increased risk of graft loss (odds ratio [OR], 2.68; 95% CI, 1.43-5.02; I^2^ = 46%; Pheterogeneity = 0.11) (Figure 4). Heterogeneity was explained by the different criteria for identifying “CD20-positive” versus “CD20-negative” biopsies included in these reports, as identified by a subgroup analysis. Three studies, including 117 patients, used a threshold cell count of more than 275 in the selected high power field (HPF) as the definition of CD20 positive. These studies showed an association between CD20+ cell infiltration during rejection and graft loss during follow up [2, 6, 7] (OR, 5.37; 95% CI, 2.25-12.78; I^2^ = 0%; Pheterogeneity = 0.53) (Figure 4). No associations were found in the analysis of studies using other threshold definitions [4, 9] (OR, 1.05; 95% CI, 0.39-2.83; I^2^ = 0%; Pheterogeneity = 0.41).

### Quality assessment results

The details of the quality evaluation, based on the Newcastle-Ottawa Quality Assessment Scale for each study, are shown in Supplementary Table 1. Two studies were considered to be of moderate quality and three of high quality. The average score was 7.6.

Although the evidence demonstrated little risk of bias, consistency, and directness and no publication bias, all of the studies included were observational studies. Thus, the overall GRADE (Grading of Recommendations Assessment, Development and Evaluation) strength of the evidence was rated as low for graft loss. The strength of the evidence for steroid resistance was increased by the very large effect, but at the same time, it was decreased by the wide confidence intervals, and was therefore rated low as well (Supplementary Table 2). Further research is required to strengthen our confidence in the estimate of the effects.

### Sensitivity analyses

The results remained unchanged after applying a random effect model or omitting each individual study.

### Publication bias

Contour-enhanced funnel plots, Begg's regression test and Egger's regression test revealed no publication bias regarding graft loss (Begg's test, *P* = 0.806; Egger's test, *P* = 280) or steroid resistance (Begg's test, *P* = 0.734; Egger's test, *P* = 0.220). The funnel plots for each meta-analysis are available in Supplementary Figures 1 and 2.

## DISCUSSION

To the best of our knowledge, the present meta-analysis is the first to explore the potential relationship between CD20+ B-cell infiltration and the outcomes of kidney graft after acute rejection. We found that the presence of CD20+ B cells was a potential cause of more aggressive and steroid-recalcitrant graft rejection. It was also associated with poor graft outcomes. This may be related to the antibody-dependent and antibody-independent roles of B cells. The latter includes the capacity to secrete inflammatory cytokines and chemokines [15–18], antigen presentation [19–21], T-cell and dendritic-cell regulation [22–26], as well as a role in lymphoid tissue development [27, 28]. Bagnasco et al. found that CD3-positive T cells and CD20-positive B cells were in the same infiltrate in some cases [9], and Hwang et al. found that the patients with CD20+ CD38+ infiltration had poorer graft prognosis compared with patients with only CD20+ infiltrates [7]. These studies indicated that the interaction of CD20+ B cells with other immune cells may account for the progress of graft inflammation. The complexity of differences in patient populations in the included studies cannot be overestimated, including different ethnicities, primary kidney diseases, surgical skill levels, immunosuppression protocols, intervals from transplantation to rejection, previous rejections and rejection etiologies. All of these factors could influence immunological processes and their downstream molecular and cellular responses. However, there was no sufficient information available in the included trials to conduct a meta-regression or subgroup analyses of these factors. In addition, a lack of standardized criteria for defining CD20-positive and CD20-negative biopsies may have caused heterogeneity among the different studies. The subgroup analyses suggested that the presence of ≥ 275 CD20+ cells /HPF was potentially a poor prognostic indicator. A difference was not revealed when other definitions of CD20 positive biopsies were used. However, taking ≥ 275 CD 20+ cells/HPF as a threshold in clinical is unwise, since only 3 studies with 117 participants proved the association. More studies with large sample size are needed to draw the conclusion.

C4d is one of the by-products of the classical complement activation pathway initiated by alloantibody production. Thus, linear C4d staining in peritubular capillaries indicates that rejection is humorally mediated [30, 31]. However, the sensitivity and specificity of C4d staining alone as a diagnostic criterion for antibody-mediated rejection (AMR) has been challenged in the Banff 2011 Meeting Report and Banff 2013 Meeting Report [32, 33]. Histological evidence of acute tissue injury, serological evidence of DSAs, and other evidence of current/recent antibody interactions are needed to identify the immune mechanism. In the present study, an association of CD20+ B cell infiltration with C4d-positive staining in biopsies from patients experiencing renal rejection biopsies was not found. However, we could not exclude the attributable role of CD20+ cell infiltration in the conventional antibody-mediated rejection. Other stronger biomarkers of humoral rejection (such as DSA) are needed to reveal the relationship.

Although our systematic review and meta-analysis indicated that CD20+ cell infiltration is a risk factor for poor graft outcomes after acute rejection, this study has several limitations that should be considered. Subtle differences in the types of rejection described in the included trials, as shown in Table 1, might have affected the result. In addition, taking overall graft loss as a prognostic indicator may not be persuasive enough, since the follow-up lengths of the studies were not identical. However, there was no sufficient graft-survival information at each time point to conduct a meta-analysis. Besides, the sample size was small, with only 200 participants evaluated, and the trials included were observational studies with low GRADE ratings. More persuasive evidence, such as that from randomized controlled trials (RCTs) with larger numbers of patients worldwide are needed. Nevertheless, the present study still verifies that the presence of CD20+ B cells in renal biopsies could be a predictive marker for worse allograft outcomes after transplant rejection.

## MATERIALS AND METHODS

### Study design

Studies that met the following eligibility criteria were included in this meta-analysis: 1) a cohort study; 2) the study participants had undergone acute rejection, defined by the Banff 1997 classification of allograft histopathology [34]; 3) they identified CD 20+ B-cell infiltration via immunohistochemical staining of renal biopsy samples from cases of rejection; and 4) the study reported the incidence of graft loss. The primary outcome was the incidence of graft loss during follow-up. The secondary outcomes were the incidence of steroid resistance and C4d staining of renal allografts during transplant rejection.

### Search strategy

The MEDLINE, EMBAS, and Cochrane Library databases were searched through January 14, 2016, with no language or regional restrictions. The following Medical Subject Heading terms and text words and their synonyms were used: allograft rejection, kidney transplantation, graft outcome, and CD20. Titles and abstracts were independently screened by two investigators (QS, RW). If insufficient information was provided in the abstract regarding the inclusion and exclusion criteria, a full-text evaluation was performed by the same two authors to determine the eligibility of the study. Discrepancies were resolved by a third author (BL).

### Data extraction

Data extraction was conducted independently by two investigators (ZC, HJ). The content extracted included the name of the first author, year of publication, follow-up length, and patient characteristics, such as age, sex, ethnicity, donor type (living or deceased), re-transplantation, PRAs, DSAs, HLA mismatches, B-FCXM, cold ischemia time, time post-transplantation, immunosuppressive therapy and outcomes of interest. Disagreements were resolved by reaching a consensus or via re-extraction of the data by a third person (YL).

The Newcastle-Ottawa Scale (NOS) Assessment for cohort studies [35] was applied to evaluate the quality of the studies by two independent reviewers (YL, BL). Three aspects were included: selection of cases and controls, comparability between them, and assessment of outcomes. Assessment of comparability was based on age, sex, ethnicity, and donor type. The total score ranged from 0 (lowest) to 9 (highest), with 5 or less deemed low quality, 6 or 7 deemed moderate quality, and 8 or 9 deemed high quality. Any discrepancies were addressed via discussion or by a third reviewer (JC).

The strength of evidence for each outcome was assessed using the GRADE guidelines [36]. The quality of evidence was rated as high, moderate, low, or very low according to the study characteristics as follow: limitations of the study design, inconsistencies, indirectness of evidence, imprecision, publication bias and other considerations.

### Data analysis

A meta-analysis was performed to estimate the differences in outcomes between CD20-positive kidney recipients and CD20-negative recipients using REVIEW MANAGER software, version 5.1 (REVIEW MANAGER, REVMAN, Copenhagen, Denmark). The ORs with 95% CIs were calculated using the Mantel-Haenszel equation in a fixed effects model. Potential heterogeneity was assessed using Cochran's Q-statistic test, with *P* < 0.1 considered statistically significant, and the I^2^ test, with values > 50% indicative of high heterogeneity. To explain the cause of heterogeneity among studies, a subgroup analysis was conducted. A sensitivity analysis was performed using a random effects model by removing each study to assess the consistency and stability of the results. A funnel plot analysis was conducted to evaluate potential publication biases using Stata software, version 11.0 (Stata Inc., College Station, TX). Publication bias was further measured using Begg's and Egger's tests, with a threshold P value of 0.1 considered statistically significant. The present study met the criteria of the Preferred Reporting Items for Systematic Reviews and Meta-Analyses (PRISMA) statement [37] (see data in Supplementary Checklist).

## SUPPLEMENTARY MATERIALS FIGURES AND TABLES




