# Supplementary material for: Effects of CD20+ B-cell infiltration into allografts on kidney transplantation outcomes: a systematic review and meta-analysis
Source: Oncotarget. 2017 Mar 15;8(23):37935–41. doi: 10.18632/oncotarget.16229 (PMC5514963; doi:10.18632/oncotarget.16229)
Supplement: Supplementary file 1 [file oncotarget-08-37935-s001.pdf]

Effects of CD20+ B-cell infiltration into allografts on kidney transplantation outcomes: a systematic review and meta-analysis

Supplementary Materials

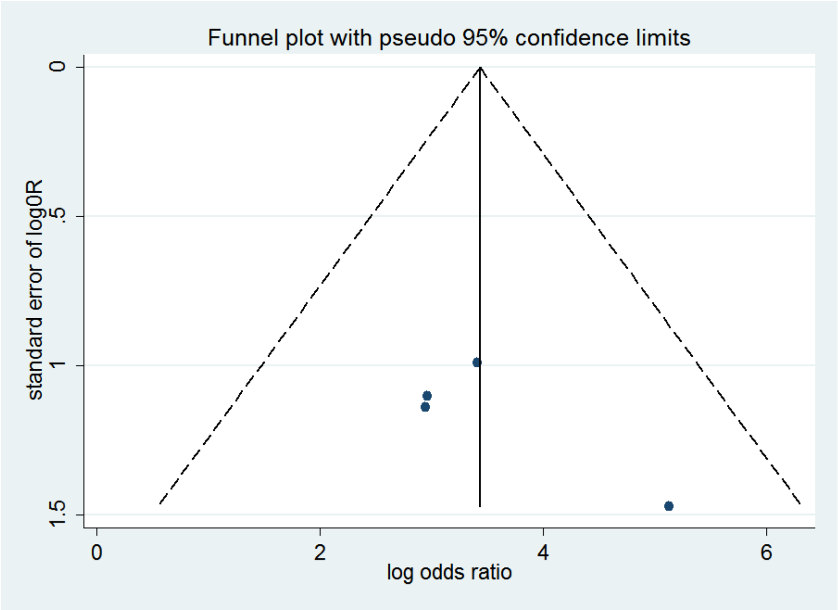

Supplementary Figure 1: Meta-analysis funnel plots of graft loss.

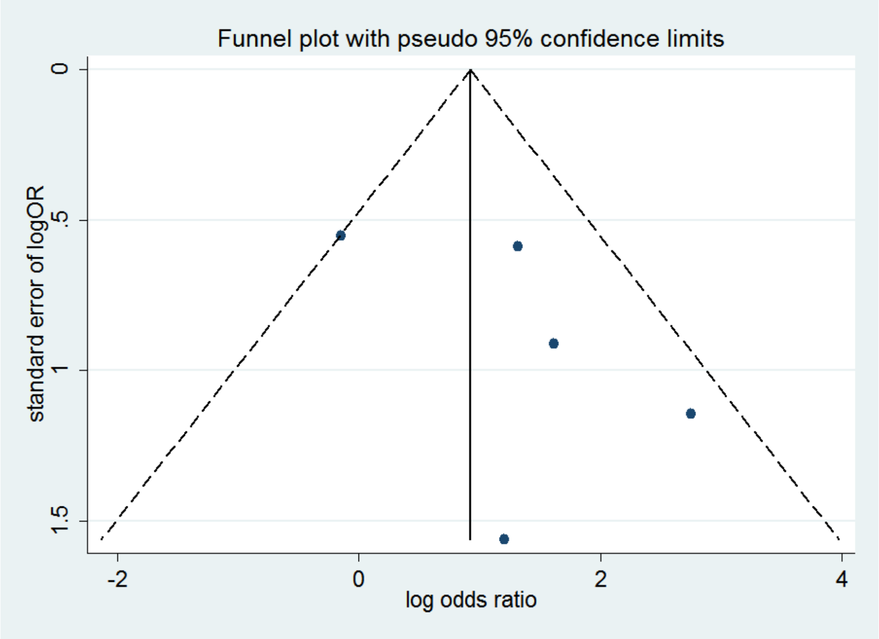

Supplementary Figure 2: Meta-analysis funnel plots of steroid resistance.

Supplementary Table 1: Quality scoring based on the Newcastle-Ottawa Quality Assessment Scale

Study, Year (Ref)	Selection	Comparability	Outcome
Bagnasco et al . 2007(11)[a]	****	*	***
Hippen et al. 2005(6)	****	**	***
Hwang et al. 2010(9)[b]	****	*	***
Minnie et al. 2003(4)	****		**
Zarkhin et al. 2008(8)[b]	****	*	***

Ref: reference; [a]: Study groups were controlled for age, gender and ethnicity in assessment of comparability, except for donor type. [b]: Study groups were controlled for age, gender and donor type in assessment of comparability, except for ethnicity.

Supplementary Table 2: Assessment of strength of recommendations using the GRADE criteria

GRADE item	Graft loss	Steroid resistance
Study limitations	Not serious	Not serious
Inconsistency	Not serious	Not serious
Indirectness	Not serious	Not serious
Imprecision	Not serious	serious
Publication bias	Undetected	Undetected
Large effect	No	Yes
Evidence of dose-response gradient	No	No
plausible confounding would change the effect	No	No
Quality of evidence	low	low

Supplementary Table 3: Examples of the potential citations that were rejected from the second screening

PMID	Cohort study	Acute rejection	IHC staining	Graft loss	CD20 positive vs CD20 negative	Reasons of been rejected
18580460	no	yes	yes	yes	yes	Review
23101480	no	yes	yes	yes	yes	RCT, not cohort study
18433412	yes	no	yes	yes	yes	recipients underwent chronic or acute on chronic allograft dysfunction, besides of acute rejection. Non-eligible population
19797166	yes	yes	no	yes	yes	Using RT-PCR, not IHC staining
17175297	yes	yes	yes	no	yes	Only Scr outcome, no graft survival outcome
17362749	yes	yes	yes	yes	no	AR vs non-AR, different group setting

IHC: immunohistochemical; RCT: randomized controlled trial; RT-PCR: reverse transcription polymerase chain reaction; Scr: serum creatinine; AR: acute rejection
